# Hair zinc levels and psychosis risk among adolescents

**DOI:** 10.1038/s41537-022-00307-y

**Published:** 2022-11-25

**Authors:** Koichi Tabata, Mitsuhiro Miyashita, Syudo Yamasaki, Kazuya Toriumi, Shuntaro Ando, Kazuhiro Suzuki, Kaori Endo, Yuko Morimoto, Yasufumi Tomita, Satoshi Yamaguchi, Satoshi Usami, Masanari Itokawa, Mariko Hiraiwa-Hasegawa, Hidehiko Takahashi, Kiyoto Kasai, Atsushi Nishida, Makoto Arai

**Affiliations:** 1 Schizophrenia Research Project, Department of Psychiatry and Behavioral Sciences, Tokyo Metropolitan Institute of Medical Science, Tokyo, Japan; 2 Department of Psychiatry and Behavioral Sciences, Graduate School of Medical and Dental Sciences, Tokyo Medical and Dental University, Tokyo, Japan; 3 Unit for Mental Health Promotion, Research Center for Social Science & Medicine, Tokyo Metropolitan Institute of Medical Science, Tokyo, Japan; 4 Department of Psychiatry, Tokyo Metropolitan Matsuzawa Hospital, Tokyo, Japan; 5 Department of Neuropsychiatry, Graduate School of Medicine, The University of Tokyo, Tokyo, Japan; 6 Department of Psychiatry, Shinshu University School of Medicine, Matsumoto, Japan; 7 Department of Psychology, Ube Frontier University, Ube, Japan; 8 Department of Computational Biology and Medical Sciences, Graduate School of Frontier Sciences, The University of Tokyo, Chiba, Japan; 9 Center for Research and Development on Transition from Secondary to Higher Education, The University of Tokyo, Tokyo, Japan; 10 Department of Evolutionary Studies of Biosystems, The Graduate University for Advanced Studies, SOKENDAI, Hayama, Japan; 11 Center for Brain Integration Research, Tokyo Medical and Dental University, Tokyo, Japan; 12 The International Research Center for Neurointelligence (WPI-IRCN) at The University of Tokyo Institutes for Advanced Study (UTIAS), Tokyo, Japan

**Keywords:** Human behaviour, Psychosis, Psychosis, Biomarkers

## Abstract

Recent meta-analyses have shown lower zinc and higher copper levels in the serum of people with schizophrenia than in healthy controls. However, the relationship between trace elements (TEs) and the pathophysiology of psychosis, including schizophrenia, remains unclear due to the antipsychotic effects on mineral levels. In this study, we aimed to determine the relationship between zinc and copper levels in hair and psychosis risk among drug-naïve adolescents. This study was conducted as a part of a population-based biomarker subsample study of the Tokyo Teen Cohort Study, including 252 community-dwelling 14-year-old drug-naïve adolescents. Zinc and copper levels in hair were measured using inductively coupled plasma mass spectrometry. The thought problems (TP) scale from the Child Behavior Checklist was used to evaluate psychosis risk. Regression analysis showed that hair zinc levels were negatively correlated with the TP scale (*T*-score) (*β* = −0.176, *P* = 0.005). This result remained significant after adjusting for age and sex (*β* = −0.175, *P* = 0.005). In contrast, hair copper levels were not associated with the TP scale (*T*-score) (*β* = 0.026, *P* = 0.687). These findings suggest that lower zinc levels could be involved in the pathophysiology of psychosis, independent of antipsychotics. Further longitudinal studies are required to investigate whether hair zinc level is a useful new biomarker for assessing psychosis risk.

## Introduction

Schizophrenia is a major mental disorder characterized by psychotic symptoms, flattening of affect, and cognitive impairment, and many people with schizophrenia do not fully recover, resulting in social and occupational dysfunction^1^. In adolescence, the prevalence of psychotic experiences (PEs) is as high as 8%^2^, and PEs are considered early indicators of psychosis, including schizophrenia^3–5^. However, the biological mechanism underlying the relationship between adolescent PEs and psychosis is unclear, and there are no established biomarkers for early intervention for psychosis. As the first step to elucidate this issue, it appears reasonable to examine whether trace elements (TEs), one of the potential biomarkers of schizophrenia, also have important roles in psychosis risk among adolescents.

Recent meta-analyses have shown significant differences in the serum levels of several TEs between people with schizophrenia and healthy controls^6,7^. Lower zinc and higher copper levels were well confirmed in schizophrenia, and this finding was also consistent in studies using hair samples^8,9^. Furthermore, these levels were significantly associated with symptomatic severity; the positive and negative syndrome scale (PANSS) scores were negatively correlated with serum and hair zinc levels^9,10^ and positively correlated with hair copper levels^8^. However, people with schizophrenia may present with different dynamics of TEs compared to drug-naïve people with first-episode psychosis (FEP) or psychosis risk due to the antipsychotic effects on mineral levels, and the mechanism underlying abnormal mineral levels in people with schizophrenia is unknown. Indeed, several studies have shown that TEs are affected by antipsychotics, including aripiprazole, risperidone, and clozapine^11–13^. In drug-naïve people with FEP, some studies showed that zinc levels were lower and copper levels did not significantly differ in serum compared to healthy controls^10,11^. However, to the best of our knowledge, no studies have been conducted on drug-naïve adolescents with psychosis risk. Therefore, the relationship between TEs and psychosis risk remains unclear.

To understand the role of TEs in the relationship between adolescent PEs and psychosis, we investigated the relationship of zinc and copper levels in hair with the thought problems (TP) scale, which is an indicator of psychosis risk among healthy adolescents^14,15^.

## Results

Table 1 shows the characteristics of the participants who were analyzed. There were no sex differences in terms of age (*χ*^2^ = 15.94, *P* = 0.938), TP scale (*T*-score) (*P* = 0.104), or hair zinc levels (*P* = 0.613). However, hair copper levels were significantly higher in females than in males (*P* = 0.002) (data not shown).

**Table 1:** Characteristics of participants.

Variables	(*N* = 252)
Age (months, mean ± SD)	178.9 ± 5.9
Sex (male/female)	134/118
TP scale^a^ (*T*-score) (mean ± SD)	58.7 ± 10.1
Hair zinc levels (ppm, mean ± SD)	150.0 ± 36.5
Hair copper levels (ppm, mean ± SD)	21.7 ± 15.7

*TP scale* thought problems scale, *ppm* parts per million, *SD* standard deviation.

^a^The TP scale is a subscale of the Child Behavior Checklist.

**Fig. 1:**
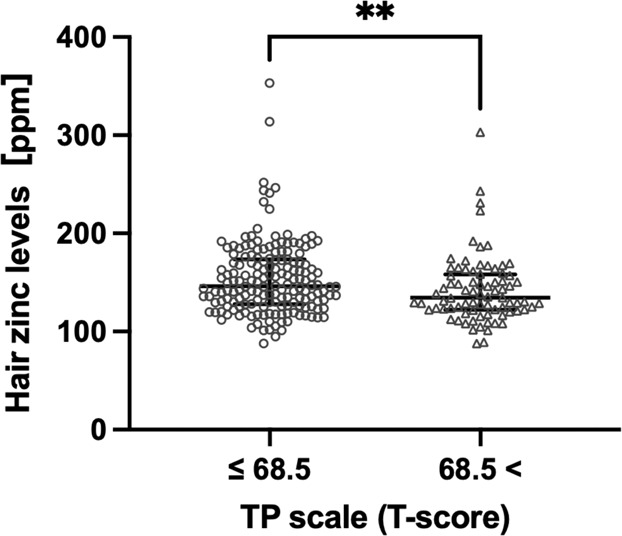
Scatterplots of hair zinc levels in participants with TP scale (*T*-score) > 68.5 and TP scale (*T*-score) ≤ 68.5. **P < 0.01. The data are presented as median with interquartile range. TP thought problems, ppm parts per million.

According to a previous study^16^, a *T*-score of the TP scale > 68.5 is considered the cut-off for psychosis. Thus, 80 participants (31.7%) were identified with possible psychosis in the present study. As shown in Fig. 1, the hair zinc levels of the 80 participants were significantly lower than those of the other 172 participants (*P* < 0.01).

**Fig. 2:**
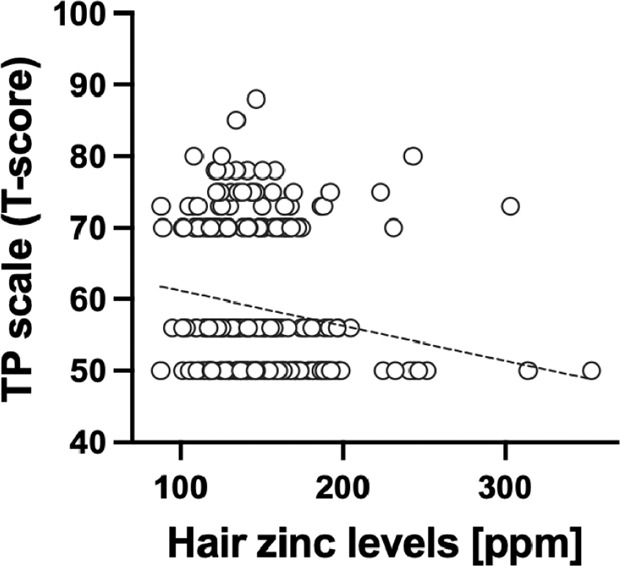
Correlation between hair zinc levels and the TP scale (*T*-score). The regression line is shown on the graph. TP thought problems, ppm parts per million.

Regression analysis showed that hair zinc levels were negatively correlated with the TP scale (*T*-score) (*β* = −0.176, *P* = 0.005) (Table 2). Figure 2 also shows the correlation of the TP scale (*T*-score) with hair zinc levels. This correlation remained significant after adjusting for age and sex (*β* = −0.175, *P* = 0.005). In contrast, hair copper levels were not associated with the TP scale (*T*-score) (*β* = 0.026, *P* = 0.687).

**Table 2:** Association between hair mineral levels and TP scale (*T*-score).

	Unadjusted model				Adjusted model			
	*β*	95% CI	*P*	Adjusted *R*²	*β*	95% CI	*P*	Adjusted *R*²
Hair zinc levels	−0.176	−0.299, −0.054	0.005^**^	0.027	−0.175	−0.297, −0.052	0.005^**^	0.028
Hair copper levels	0.026	−0.099, 0.150	0.687	−0.003	0.047	−0.081, 0.176	0.470	−0.001

*TP scale* thought problem scale, *CI* confidence interval.

**P < 0.01.

^a^Adjusted for age and sex.

Using the interquartile range (IQR), five outliers for the TP scale and four outliers for hair zinc levels were identified. The IQR was defined as the difference of the third quartile (Q_3_) minus the first quartile (Q_1_) of distribution, and these outliers were higher than Q_3_ plus 1.5 times the IQR. There were no outliers that were lower than Q_1_ minus 1.5 times the IQR. Regression analysis after removing these outliers also showed that hair zinc levels were negatively correlated with the TP scale (*T*-score) (*β* = −0.211, *P* = 0.001). This correlation remained significant after adjusting for age and sex (*β* = −0.212, *P* = 0.001) (data not shown).

## Discussion

Hair zinc levels were negatively associated with the TP scale among drug-naïve adolescents, suggesting that lower zinc levels could be involved in the pathophysiology of psychosis independent of antipsychotics. In contrast, hair copper levels were not associated with psychosis risk. These findings are consistent with recent meta-analyses that showed lower zinc levels in people with schizophrenia^6,7^. Interestingly, our findings are also consistent with previous studies of drug-naïve people with FEP, in which zinc levels were lower, and copper levels did not significantly differ in the serum compared to healthy controls^10,11^. Zinc may play an important role in the development of psychosis. In contrast, higher copper levels in people with schizophrenia may indicate antipsychotic effects.

Preceding studies showed that the increase in hair zinc levels began in later childhood and continued until adolescence, before decreasing over time^17^ and that the peak zinc levels were generally observed between adolescence and adulthood^18,19^. All the participants in this study were adolescents, indicating that a deviation from the normal trajectory of hair zinc levels may be involved in the development of psychosis. In addition to previous findings showing an association of lower zinc levels with chronic schizophrenia^6,7^ or FEP^10,11^, drug-naïve adolescents with psychotic symptoms also showed lower hair zinc levels. These findings suggest that hair zinc levels may be a new biomarker of psychosis risk in a population with wide-ranging ages. However, further longitudinal studies are required to examine the utility of hair zinc levels as a biomarker of psychosis.

Several explanations could be considered for the association between lower zinc levels and the pathophysiology of psychosis risk. First, zinc is an essential TE in numerous biological processes^20^ and has the highest concentration in the brain compared to other organs in the human body^21^. Zinc also serves as an endogenous neuromodulator of several important glutamate receptors, including N-methyl-d-aspartate (NMDA) receptors, γ-amino butyric acid receptors, and the α-amino-3-hydroxy-5-methyl-4-isoxazole-propionic acid/kainate receptors^22,23^. In zinc-deficient young rats, increased extracellular glutamate was observed in the hippocampus^23,24^. In the cortical synaptic membranes of guinea pigs, zinc deficiency decreased the concentration of NMDA receptors^25^. Notably, these findings are consistent with the glutamate hypothesis for schizophrenia^26^. Second, zinc is a component of more than 300 enzymes, and it is involved in systemic physiology, including antioxidant and anti-inflammatory effects^21^. In in vivo and in vitro cell culture experiments, zinc deficiency was associated with increased oxidative (thiobarbituric acid-reactive substances and protein carbonyl content) and inflammatory (IL-1β, IL-6, and TNF-α) factors^27,28^. Recently, the relationship between these factors and the pathophysiology of schizophrenia has been confirmed in human studies^29,30^. Thus, lower zinc levels may affect psychosis development by inducing glutamate dysfunction, oxidative stress, and inflammation.

It remains unclear how hair zinc levels were lowered among adolescents at psychosis risk. One possible explanation is that genetic variations in zinc homeostasis may alter their early neurodevelopment. Zinc homeostasis is maintained by metallothioneins (MTs)^31^ and two families of zinc transporters: ZnTs (Zn transporters) and ZIPs (Zrt- and Irt-like proteins)^32^. Recent genome-wide association studies have revealed that a single nucleotide polymorphism in zinc homeostasis genes (*ZnT3*, *ZIP8*, and *ZIP12*) is related to the risk of schizophrenia^33–35^. Furthermore, schizophrenia-like behavioral disturbances, including reduced pre-pulse inhibition and deficits in learning, memory, and social interactions, were observed in the animal models with a targeted disruption of zinc homeostasis genes (*ZnT3* and *MT3*)^36,37^. Thus, further studies are required to clarify the effect of genetic heterogeneity in zinc homeostasis on psychosis risk.

Our findings suggest important clinical implications for the early identification and intervention of psychosis. In clinical and community settings, it could be recommended that adolescents with lower hair zinc levels receive zinc supplementation, dietary guidance, and psychosocial support. Indeed, a double-blind, randomized, placebo-controlled trial in people with schizophrenia showed that zinc sulfate add-on therapy to risperidone significantly decreased the PANSS scores compared to risperidone monotherapy^38^.

We have discussed the association between zinc and psychosis, particularly in schizophrenia. However, zinc may be widely involved in the pathophysiology of other mental disorders due to its role in numerous biological processes^20^. Some meta-analyses have shown that lower zinc levels were also observed in people with depression^39^ and neurodevelopmental disorders, including autism spectrum disorder^40^ and attention-deficit hyperactivity disorder^41^. Thus, further studies in adolescents will be required to investigate the relationship between lower zinc levels and the risk of these disorders.

## Strengths and limitations

This study had several strengths. First, adolescents without antipsychotic medications participated in the study, which allowed us to exclude the antipsychotic effects on mineral levels. Therefore, our findings suggest the essential role of TEs in the pathophysiology of psychosis. Second, using a few strands of hair to measure stable mineral levels was less invasive than the collection of blood samples.

This study had some limitations. First, we did not conduct medical interviews, including the structured interview for prodromal syndromes (SIPS) and the comprehensive assessment of ARMS (CAARMS), but used the TP scale, which has only seven items and was evaluated by the primary caregivers. Although previous studies showed an association between the TP scale and psychosis risk^14,15^, they may have underestimated or overestimated the children’s abnormal behaviors, and this observer bias was not considered in the present study. Second, regression analysis showed that hair zinc levels were significantly correlated with the TP scale (*T*-score); nonetheless, the adjusted *R*^2^ in the analysis was low, indicating that further validation is required regarding the use of hair zinc levels as a clinical biomarker. Additionally, the study design was cross-sectional and the sample size was not large although participants were recruited from a large-scale population-based birth cohort study. Therefore, further longitudinal studies with larger samples size are required to confirm the causal relationship between hair zinc levels and psychosis development. Third, we could compare the TP scale between adolescents at risk of psychosis and people with psychosis using internationally standardized *T*-scores. However, regarding the hair mineral levels, we could not compare psychosis risk with psychosis because we could not obtain the hair samples from individuals with psychosis, and measurement methods and standard values differ between studies. In the future, it will be necessary to investigate the difference in hair mineral levels between adolescents with psychosis risk and psychosis.

## Conclusion

In a population-based birth cohort study of adolescents, lower hair zinc levels could be associated with psychosis risk among drug-naïve adolescents, suggesting its involvement in the pathophysiology of psychosis, independent of antipsychotics. Further longitudinal studies are required to investigate hair zinc level as a new biomarker for assessing psychosis risk.

## Methods

### Participants

**Fig. 3:**
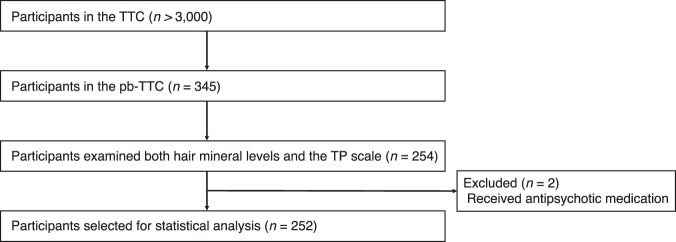
Participant flowchart. TTC the Tokyo Teen Cohort Study, pb-TTC the population-based biomarker subsample study of the Tokyo Teen Cohort Study, TP thought problems.

This study was conducted as part of the population-based biomarker subsample study of the Tokyo Teen Cohort Study (pb-TTC), which was a large-scale population-based birth cohort study conducted in the Tokyo Metropolitan area with >3000 adolescent-caregiver dyads (TTC, http://ttcp.umin.jp/). The pb-TTC included 345 adolescents (mean [SD], 13.5 [0.6] years), of which 282 adolescents participated in a second survey at age 14, and both hair mineral levels and the TP scale were examined in 254 adolescents. Two adolescents received antipsychotic medication during the study, and the other 252 adolescents were finally selected for statistical analysis to exclude the effect of antipsychotics on mineral levels (Fig. 3).

Written informed consent was obtained from each participant and their primary caregiver before participation. The research ethics committee of the Tokyo Metropolitan Institute of Medical Science approved this study.

### Measurement of hair mineral levels

Serum mineral levels fluctuate with diurnal variation and dietary intake^42^. However, hair mineral levels stably reflect the accumulation of TEs in the human body over months to years^43^ and are confirmed as the best indicators of mineral levels in the body^44^. Furthermore, hair sampling is a less invasive measurement than blood collection.

Hair samples (~3 cm in length and weighing 0.1 g) were obtained from each participant, with the hair cut close to the scalp. The samples were sent to the La Belle Vie research laboratory, and mineral levels (zinc and copper) were measured using the methods as described below^45^.

Hair samples (75 mg) were weighed into 50 mL plastic tubes and washed twice using acetone and then with 0.01% Triton solution, in accordance with the procedures recommended by the Hair Analysis Standardization Board^46^. The washed hair samples were mixed with 10 mL 6.25% tetramethylammonium hydroxide (TMAH, Tama Chemical) and 50 µL of 0.1% gold solution (SPEX Certi Prep.) and then dissolved at 75 °C with shaking for 2 h. After cooling the solution to room temperature, an internal standard (Sc, Ga, and In) solution was added. The volume was adjusted gravimetrically, and the obtained solution was used for mineral analysis. The mineral concentrations were measured with inductively coupled plasma mass spectrometry (Agilent-7500ce) using the internal standard method^47–49^ and are expressed as ng/g hair (ppb). For quality control of the mineral analysis, a certified human hair reference material supplied by the National Institute for Environmental Studies of Japan (certified reference material no. 13) was used^50^.

### Assessment of psychosis risk

The Child Behavior Checklist (CBCL) is a simple questionnaire tool developed by Achenbach that comprehensively evaluates children’s emotional and behavioral problems^51^. CBCL is widely used to predict mental disorders meeting Diagnostic and Statistical Manual of Mental Disorders criteria^52–54^, and the TP scale, a subscale of CBCL, has been shown to be an indicator of psychosis risk among adolescents^14,15^.

The TP scale was completed by caregivers (mainly mothers) and included the following seven items: (i) ‘Can’t get his/her mind off certain thoughts; obsessions’, (ii) ‘Hears sounds or voices that aren’t there’, (iii) ‘Repeats certain acts over and over; compulsions’, (iv) ‘Sees things that aren’t there’, (v) ‘Stares blankly’, (vi) ‘Strange behavior’ and (vii) ‘Strange ideas’^51,55^. All the responses were rated on a 3-point scale: not true = 0, somewhat/sometimes true = 1, and very true/often true = 2. The TP scale was defined as the total score of the seven items (possible range: 0–14). The standard values of CBCL vary according to country^56,57^. As such, TP scores were converted into *T*-score based on Japanese standard values^55^ for international comparisons.

### Statistical analysis

Data are presented as the mean [SD]. Non-parametric analyses using the Mann–Whitney *U* test or *χ*^2^ test were performed to examine sex differences and compare hair zinc levels between participants with TP scale (*T*-score) > 68.5 and TP scale (*T*-score) ≤ 68.5. The threshold for significance was defined as *P* < 0.05. Regression analysis was used to examine the correlation between hair mineral levels (zinc and copper) and the TP scores among adolescents. Age and sex were covariates due to their possible correlation with the TP scale^51,55^. The significance level (*α*) was set to 0.05 for two-tailed tests. All statistical analyses were performed using IBM SPSS for Mac OS, version 26.0 (IBM Corp., New York, USA).

## Data Availability

The data described in the manuscript, code book and analytic code will be made available upon request pending.
